# Adherence to Self-Administered Tuberculosis Treatment in a High HIV-Prevalence Setting: A Cross-Sectional Survey in Homa Bay, Kenya

**DOI:** 10.1371/journal.pone.0032140

**Published:** 2012-03-12

**Authors:** Fabienne Nackers, Helena Huerga, Emmanuelle Espié, Apollo Odongo Aloo, Mathieu Bastard, Jean-François Etard, Joseph Sitienei, Francis Varaine, Jeremiah Chakaya, Maryline Bonnet

**Affiliations:** 1 Epicentre, Paris, France; 2 Médecins Sans Frontières-France, Paris, France; 3 National Leprosy Tuberculosis Program, Nairobi, Kenya; 4 IRD/UMI 233 TransVIHMI, Montpellier, France; 5 Centre for Respiratory Diseases Research, Kenyan Medical Research Institute, Nairobi, Kenya; UCL Institute of Child Health, University College London, United Kingdom

## Abstract

**Background:**

Good adherence to treatment is crucial to control tuberculosis (TB). Efficiency and feasibility of directly observed therapy (DOT) under routine program conditions have been questioned. As an alternative, Médecins sans Frontières introduced self-administered therapy (SAT) in several TB programs. We aimed to measure adherence to TB treatment among patients receiving TB chemotherapy with fixed dose combination (FDC) under SAT at the Homa Bay district hospital (Kenya). A second objective was to compare the adherence agreement between different assessment tools.

**Methods:**

We conducted a cross-sectional survey amongst a series of new TB patients receiving 6 months of standard TB chemotherapy with FDC under SAT. Adherence was assessed at home with urine testing for Isoniazid (INH), pill count, interviewer-administered questionnaire and visual analogue scale (VAS).

**Results:**

In November 2008 and in June 2009, 212 of 279 eligible patients were assessed for adherence. Overall, 95.2% [95%CI: 91.3–97.7] of the patients reported not having missed a tablet in the last 4 days. On the VAS, complete adherence was estimated at 92.5% [95%CI: 88.0–95.6]. INH urine test was positive for 97.6% [95%CI: 94.6–99.2] of the patients. Pill count could be assessed among only 70% of the interviewed patients. Among them, it was complete for 82.3% [95%CI: 75.1–88.1]. Among the 212 surveyed patients, 193 (91.0%) were successfully treated (cured or treatment completed). The data suggest a fair agreement between the questionnaire and the INH urine test (k = 0.43) and between the questionnaire and the VAS (k = 0.40). Agreement was poor between the other adherence tools.

**Conclusion:**

These results suggest that SAT, together with the FDC, allows achieving appropriate adherence to antituberculosis treatment in a high TB and HIV burden area. The use of a combination of a VAS and a questionnaire can be an adequate approach to monitor adherence to TB treatment in routine program conditions.

## Introduction

Good adherence to tuberculosis (TB) treatment is crucial to cure patients, to limit the development of drug resistance and to reduce TB transmission in the community. For years, WHO has been recommending the administration of drugs through directly observed therapy (DOT) as part of the control strategy called DOTS [1]. The efficiency and feasibility of DOT in routine health care programs have been questioned for several reasons: i) DOT requires well functioning and well staffed health services which may not be available in some high burden and limited resource countries [2], [3]; ii) DOT is expensive, and time-consuming for patients [4]; iii) the appropriateness of using DOT for TB treatment in regions of high HIV prevalence where antiretroviral treatments (ART) are self-administered may be questioned; iv) DOT has not consistently been shown to be superior to other approaches such as self-administered treatment (SAT) when comparing cure or treatment completion rates [5]; v) DOT may raise ethical issues regarding privacy and stigmatisation [6], [7]. The use of community DOT, if well monitored and supervised, can solve some of these challenges [8]. Alternatively, Médecins sans Frontières (MSF) has implemented SAT in several TB programs.

To ensure good adherence to TB treatment in these SAT based programs, MSF promotes the use of fixed dose combinations (FDC). FDCs, by considerably reducing the number of pills to swallow, are likely to enhance adherence to treatment [9]–[11]. In addition, FDCs may prevent the emergence of drug resistance and have shown similar treatment outcomes as compared to separately administered drugs [12]. The SAT approach should be associated with patients-centred adherence strategies, including continuous patient education and counselling, an adequate therapeutic environment with a patient-health care provider relationship based on trust, respect, and involvement of the patient in his/her treatment, as well as social support when necessary.

Regular adherence monitoring is essential to follow the quality of SAT based TB programs. To date, few data have been reported on adherence in such programs located in limited resource, high HIV-TB burden settings [13].

Adherence monitoring is however a challenge due to the lack of reliable tools [14]. The available tools include questionnaires, visual analogue scales (VAS), urine tests for isoniazid (INH), pill counts, and monitoring of pill collection regularity. All have limitations and usually cover different treatment intake periods. It is therefore recommended to combine tools in order to obtain a reliable and valid estimate of patient adherence [15]. Although some of these tools have been well evaluated for adherence to antiretrovirals in HIV infected patients [16]–[22] and some of these results could be extrapolated to TB patients, further evaluation in TB is necessary.

The primary objective of this study was to measure adherence to TB treatment among patients receiving 6 months of standard TB chemotherapy with FDC under SAT in a limited resource, high TB-HIV burden setting. A secondary objective was to compare the agreement between different adherence assessment tools.

## Materials and Methods

### Study setting

The study was conducted in Homa Bay district, Nyanza province, in western Kenya. This area encompasses 360,000 inhabitants. The estimated TB incidence in Kenya was 305/100,000 inhabitants in 2009 [23]. The HIV prevalence was 15.3% in Nyanza province [24] and 24% in Homa Bay District [25] where 74% of TB cases were HIV infected [26]. Since 2000, MSF has been running a medical HIV/AIDS programme in Homa Bay and has also been supporting the Ministry of Health TB clinic of the district hospital. Newly diagnosed TB patients were receiving a 6 months of standard TB chemotherapy (fixed dose combination of 2 months rifampicin (R), isoniazid (H), pirazinamide (Z) and ethambutol (E), followed by 4 months of RH), using a self-administrated approach. Patients collected the drugs weekly for the first 2 months (intensive phase), and monthly for the last 4 months (continuation phase). Patients received the exact number of pills necessary to cover the period between two visits at the clinic. Treatments strategies were based on patient-centered support and patients' education. This individual-based approach relied on a trusting relationship between care providers and patients. In practice, health education was provided as a group session by an “exemplary patient” to patients in the waiting area. Individual adherence counselling was also given by a counselor at the time of TB diagnosis and at each follow-up visit. Counselors were available to provide specific in-depth patient support according to the needs. In addition, food supplementation was given to TB patients with a body mass index under 17.5 kg/m^2^. Provider initiated voluntary counselling and testing for HIV infection was also systematically offered to all TB patients. None of the component of these SAT strategy was changed for the purpose of this study that aimed to evaluate adherence under routine program conditions.

### Study design and participants

We conducted a cross-sectional survey. Eligible patients were new TB patients, aged at least 18 years, living in Homa Bay District, with active pulmonary (both smear positive and negative) or extra pulmonary TB and receiving a fixed dose combination of 2RHZE/4RH under SAT. Patients in prison were excluded as well as patients with history of previous TB treatment (including relapse, failure and return after default) because their treatment regimen was not only based on FDC. We aimed to recruit all patients who started treatment in the 6 months preceding the start of the survey. These patients were identified through the TB register of the TB clinic of the Homa Bay district hospital and medical records. Patients who died or defaulted (interruption of treatment for two consecutives months or more) before the time of the survey could not be assessed for adherence. Also, patients hospitalized at the time of the survey were not assessed for adherence because their treatment regimen was not based only on FDC under SAT at the hospital.

Consent for an unplanned home visit was asked to all eligible patients when they presented at the TB clinic for a regular weekly or monthly visit. The purpose of the unplanned home visit was not explained. Patients who did not accept a home visit were secondarily excluded. For patients accepting, information about the survey was given at the patient's home. Patients signing the informed consent were included in the study and adherence was assessed. A second home visit was conducted in case of absence of the patient. If the patient was still absent at the second visit, he/she was recorded as “absent”.

### Adherence assessment and data collection

Recent adherence (last 4 days) and adherence during the last month were measured using simultaneously two subjective (questionnaire and VAS), and two objective adherence monitoring tools (urine test for INH and a pill count).

Self-reported recent adherence was measured by a standardised interviewer-administered questionnaire. Patients were asked to report the number of antituberculosis pills they took the day before the survey as well as 2 days, 3 days and 4 days before the survey. This number of pills was compared to number of pills prescribed to the patient. The adherence to TB medication in the last 4 days was classified as unsatisfactory (more than 25% of the pills missed in the last 4 days, corresponding to more than one daily dose missed), satisfactory (no more than 25% of the pills missed in the last 4 days, corresponding to a maximum of one daily dose missed) or complete (no missed pill in the last 4 days).

The patient's adherence to TB medication in the last month was assessed using a 10 points linear VAS (“how much of your prescribed TB medications have you taken in the last month?”). The adherence measured by the VAS was classified as unsatisfactory (<80%, that is rating a value lower than 8 on the VAS), satisfactory (≥80% but less than 100%) or complete (100%). The cut-off of 80% refers to the threshold used to define compliance in the IUAT trial of various durations of INH preventive therapy for TB [27]. The questionnaire and the VAS were pre-tested before the survey.

The questionnaire also included questions on socio-demographic characteristics, reasons for non-adherence, and adherence secondary endpoints (number of appointments missed, last time when the patient missed a pill, the way patients followed the medical prescription in the last month and a VAS on patient self-confidence to successfully take the medication).

Pill count was calculated by comparing remaining pills (missed or not yet taken), shown by the patient at home, and pills given to the patient at the last visit at the TB clinic. As the exact total number of pills delivered to the patient at the TB clinic was not always properly recorded, the number of pills received by the patient was calculated based on the prescription and the number of days between the last visit to the clinic and the day of assessment. Usually patients received treatment for 7 days during the intensive phase and 28 days during the continuation phase. The calculated proportion of pills actually taken by the patient was classified as unsatisfactory (<80%), satisfactory (≥80% but less than 100%) or complete (100%) [27].

BBL™ Taxo™ INH urine test strip was used to identify the presence of INH in the patient urine. Results were classified as positive or negative. The test has a good sensitivity to detect if INH was ingested in the previous 24 to 36 hours. Some patients may remain positive at 60 hours (28% positive) and 72 hours (4%) after drug ingestion [28].

Adherence assessments was performed by eight teams that included one medical and one non-medical person. The surveyors were not part of the staff providing care to the patient. All teams were trained in the study procedures. The questionnaire and the VAS were completed first and subsequently, patients were asked to present their drugs container and the remaining pills were counted. Urine was collected at the end for INH testing.

Patients' TB treatment outcomes were collected from the TB register at the TB clinic. The outcomes were classified following the standard WHO definitions [29].

### Sample size and statistical analysis

To measure the proportion of patients with complete adherence with a 10% precision on the estimate, we needed to recruit 81 patients considering an expected 70% complete adherence rate during the intensive phase and 97 patients considering an expected 50% adherence rate during the continuation phase (α = 0.05). Expecting about one third of the patients in the intensive phase of treatment, we needed to include a series of 243 patients.

For each adherence measurement tool, the proportions of patients with complete, satisfactory and unsatisfactory adherence were calculated. The proportions of patients with complete adherence were presented with exact 95% confidence intervals (95%CI).

Agreement between adherence measurements was assessed using the kappa coefficient. The maximum attainable kappa, that is the maximum value of the kappa coefficient attainable for this set of data and the prevalence index were also calculated [30]. Because there is no gold standard tool to measure the adherence to TB treatment, a latent class analysis (LCA) was used to estimate the true prevalence of satisfactory adherence and to assess the posterior probability of being adherent [31], [32]. LCA assumes that patients can be classified in two groups: adherent and non-adherent and allows computing the probability of being adherent for each combination of adherence measurement tools.

Data were analysed using Stata® 9 software (College Station, Texas, USA).

### Ethics

The study was approved by the scientific and ethical review committees of the Kenyan Medical Research Institute (KEMRI) in Nairobi and the Committee de Protection des Personnes de St Germain en Layes, France.

Written informed consent was obtained from each patient before participation.

Patients with poor adherence were offered additional adherence support and counselling.

## Results

### Participants

**Figure 1:**
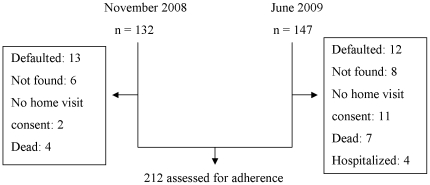
Survey Profile.

In order to obtain the estimated sample size, the survey was performed in two stages: November 2008 and June 2009. All eligible patients who started TB treatment between May and October 2008 (n = 132) were investigated in November 2008 and patients who started TB treatment between December 2008 and June 2009 (n = 147) were investigated in June 2009. There was no statistical evidence for a difference in the characteristics of the two series of patients (data not shown). Of the 279 eligible patients, 67 were not interviewed: 25 (9.0%) had defaulted prior to asking consent for a home visit, 14 could not be located (5.0%), 13 (4.7%) did not consent for a home visit, 11 (3.9%) were dead and 4 (1.4%) were hospitalised by the time of the survey (Figure 1). Socio-demographic and medical characteristics of the 212 patients included in the adherence assessment are presented in Table 1. One third (73/212) of the patients where helped by another person for treatment intake. About half of these people (56.5%) were present when the patient was answering the questionnaire.

**Table 1:** Baseline characteristics of the participants.

n (%)		Total
		(n = 212)
***Socio-demographic characteristics***		
Mean age (years) ± standard deviation		35.0±11.9
Female		98 (46.2)
Education:	Never went to school	10 (4.7)
	Incomplete primary	76 (35.9)
	Complete primary	52 (24.5)
	Incomplete secondary	24 (11.3)
	Complete secondary	32 (15.1)
	Higher level	18 (8.5)
Distance home-clinic (km):	0-<5 km	114 (55.9)
	5-<10 km	48 (23.5)
	10 km or more	42 (20.6)
Residence area:	Rural	102 (48.6)
	Urban	108 (51.4)
***Medical characteristics***		
Pulmonary tuberculosis		167 (78.8)
Weeks between the start of the treatment and the survey:		
	Median (minimum – maximum)	12.7 (0.9–24)
	1 to 7 (intensive phase)	64 (30.2)
	8 to 24 (continuation phase)	148 (69.8)
HIV Positive (n = 202)*		153 (75.7)
HIV positive under ART (n = 103)**		75 (72.8)

*10 missing data.

**ART data missing for 50 HIV positive.

### Adherence

Adherence was assessed in a median delay of 2 days after the last visit at the TB clinic (min-max: 0–7) for patients in the intensive phase of treatment, and of 8 days (min-max: 0–41) for patients in the continuation phase of treatment.

The estimated level of adherence was high using all the adherence measurement tools (Tables 2 and 3). Overall, 95.2% [95%CI: 91.3 to 97.7] of the patients reported not having missed a pill in the last 4 days. On the VAS, complete adherence was estimated at 92.5% [95%CI: 88.0 to 95.6]. Urine INH test was positive for 97.6% [95%CI: 94.6 to 99.2] of the patients. Pill count could only be assessed among 70% of the interviewed patients. The proportion of pills taken by the patient was classified as complete for 82.3% [95%CI: 75.1 to 88.1]. Except for the VAS, main adherence results tended to be better during the intensive phase than during the continuation phase. The differences were not statistically significant although there was a trend for the pill count (Table 2). There was no significant difference in adherence neither between HIV infected and non infected patients nor between patients on ART and patients not on ART (data not shown).

**Table 2:** Adherence (main endpoints) of the participants according to the phase of treatment (n = 212).

		Intensive	Continuation	p*	All
		n (%)	n (%)		n (%)
Adherence according to questionnaire**				0.40	
	Complete (no missed pill last 4 days)	61 (96.8)	137 (94.5)		198 (95.2)
	Satisfactory (max 25% missed last 4 days)	2 (3.2)	4 (2.8)		6 (2.9)
	Unsatisfactory (>25% missed last 4 days)	0 (0.0)	4 (2.8)		4 (1.9)
VAS: “how much of your prescribed TB medications have you taken in the last month”				0.84	
	Complete (100%)	59 (92.2)	137 (92.6)		196 (92.5)
	Satisfactory (≥80%)	5 (7.8)	10 (6.8)		15 (7.0)
	Unsatisfactory (<80%)	0 (0.0)	1 (0.6)		1 (0.5)
INH test	Positive	64 (100)	143 (96.6)	0.33	207 (97.6)
	Negative	0 (0.0)	5 (3.4)		5 (2.4)
Pill count***				0.08	
	Complete (100%)	49 (90.7)	72 (77.4)		121 (82.3)
	Satisfactory (≥80%)	2 (3.7)	14 (15.1)		16 (10.9)
	Unsatisfactory (<80%)	3 (5.7)	7 (7.5)		10 (6.8)

*Fisher exact.

**4 missing data.

***65 missing data or inconsistencies.

**Table 3:** Adherence (secondary endpoints) of the participants according to the phase of treatment (n = 212).

	Intensive	Continuation	p*	All
	n (%)	n (%)		n (%)
≥1 appointment(s) missed for drug collection	0 (0.0)	5 (3.4)	0.33	5 (2.4)
Last time reported to have missed a pill**			0.84	
Never missed a pill	55 (93.2)	128 (90.1)		183 (91.0)
Last 4 days	2 (3.4)	5 (3.5)		7 (3.5)
More than 4 days ago	2 (3.4)	9 (6.3)		11 (5.5)
Regarding your TB treatment, in the last month, how did you follow the medical prescription:			0.60	
Strictly	59 (95.1)	143 (96.6)		202 (96.2)
Approximately with few deviations	3 (4.8)	4 (2.7)		7 (3.3)
With many deviations	0 (0.0)	1 (0.7)		1 (0.5)
Rarely/Did not take any pill	0 (0.0)	0 (0.0)		0 (0.0)
Never stop taking pill for more than 2 days	60 (95.2)	136 (93.8)	1.0	196 (94.2)
VAS: “how confident do you feel you can successfully take your medication”			0.001	
100%	56 (87.5)	143 (96.6)		199 (93.9)
≥80%	8 (12.5)	2 (1.4)		10 (4.7)
<80%	0 (0.0)	3 (2.0)		3 (1.4)

*Fisher exact.

**11 missing data.

For 16 patients (7.5%), adherence was classified as unsatisfactory by at least one of the 4 tools. Taking in consideration the 4 tools and excluding patients with missing data for any one of the 4 tools, 91.1% [133/146; 95%CI 85.3–95.2] of patients interviewed had a satisfactory adherence on all the 4 adherence measurements tools (INH test positive, VAS≥80%, questionnaire ≥75%, pill count ≥80%). The LCA model estimated the prevalence of true satisfactory adherence at 99% [95%CI 97–100%] . If the 25 patients who defaulted before being asked consent for a home visit were included in the estimation of the adherence level and considering that all of them would have had a non-satisfactory adherence, the overall adherence would have been 77.8% (133/171) [95%CI 70.8–83.8].

Reasons for non-adherence were reported by 17 patients who had missed at least one pill through the questionnaire and/or the VAS. The main reasons were: running out of pills (22%), being away from home (19%) or forgetting to take the medication (17%).

Among the 212 surveyed patients, 193 (91.0%) were successfully treated (cured or treatment completed) (Table 4). Among the 279 eligible patients, the success rate was 78.9%. The proportion of unfavourable outcomes (death, failure, or default excluding patients transferred out) tended to be lower in the group of patients with satisfactory adherence (13/192, 6.8%) than in the group of patients with unsatisfactory adherence by at least one of the 4 tools (2/16, 12.5%; p = 0.32) but the difference was not statistically significant.

**Table 4:** TB treatment outcome.

n (%)	Patients surveyed	All eligible patients
	(n = 212)	(surveyed or not; n = 279)
Success*	193 (91.0)	214 (78.9)
Death	6 (2.8)	19 (7.0)
Failure	4 (1.9)	4 (1.5)
Default	5 (2.4)	30 (11.1)
Transferred out	4 (1.9)	4 (1.5)
Missing data	/	8

* Cured or treatment completed.

### Tools agreement

The data suggest a fair agreement between the questionnaire and the INH urine test (k = 0.43) and between the questionnaire and the VAS (k = 0.40) (Table 5). Agreement was poor between the other adherence tools (k<0.40). Due to the high number of missing values, the pill count was not included in the LCA model. According to the LCA model, having an adherence classified as satisfactory by at least two of the three tests (INH and/or VAS and/or questionnaire) gave a posterior probability of being satisfactory adherent equal to 1 (Table 6). Posterior probability of being satisfactory adherent with an adherence classified as satisfactory by only one of the three tests was inferior or equal to 0.5. Predicted frequencies by the model show the good fit of the data.

**Table 5:** Agreement between adherence measurement tools.

	Questionnaire <75%	Questionnaire ≥75%	Total
**VAS <80%**	1	0	1
**VAS ≥80%**	3	204	207
Total	4	204	208
*k: 0.40*	*Maximum attainable k: 0.40*	*Prevalence index: 0.98*	

	Questionnaire <75%	Questionnaire ≥75%	
**INH test −**	2	3	5
**INH test +**	2	201	203
Total	4	204	208
*k: 0.43*	*Maximum attainable k: 0.87*	*Prevalence index: 0.96*	

	Questionnaire <75%	Questionnaire ≥75%	
**Pill count <80%**	1	9	10
**Pill count ≥80%**	1	135	136
Total	2	144	146
*k:* 0.15	*Maximum attainable k: 0.32*	*Prevalence index: 0.92*	

	VAS <80%	VAS ≥80%	
**INH test −**	1	4	5
**INH test +**	0	207	207
Total	1	211	212
*k: 0.33*	*Maximum attainable k: 0.33*	*Prevalence index: 0.97*	

	VAS <80%	VAS ≥80%	
**Pill count <80%**	1	9	10
**Pill count ≥80%**	0	137	137
Total	1	146	147
*k: 0.17*	*Maximum attainable k: 0.17*	*Prevalence index: 0.93*	

	INH test −	INH test +	
**Pill count <80%**	1	9	10
**Pill count ≥80%**	2	135	137
Total	3	144	147
*k: 0.13*	*Maximum attainable k: 0.44*	*Prevalence index: 0.91*	

**Table 6:** Posterior probability of adherence (latent class analysis).

VAS	Questionnaire	INH test	Probability of being adherent
+	+	+	1
+	+	−	1
+	−	+	1
+	−	−	0.03
−	+	+	1
−	+	−	0.01
−	−	+	0.51
−	−	−	0

+: complete or satisfactory adherence.

−: unsatisfactory adherence.

## Discussion

The results of this study suggest a good adherence to the self-administered TB treatment with FDCs for new TB cases in Homa Bay district, western part of Kenya. Both recent adherence (measured through questionnaire and INH test) and last month adherence (measured through VAS) to TB treatment were good. There was no evidence for a difference in adherence during intensive and continuation phase of the TB treatment. Similar good adherence has been previously reported using SAT in a slum in Nairobi [13] and experiences from various countries have shown that interventions such as enabling patients to take responsibility for their health or increasing flexibility in terms of patient choice of treatment strategy, could improve adherence to TB treatment [33], [34]. The main reason of non adherence reported by the patients was running out of pills, as reported in other African settings [35]. The reason for running out of pills was likely to be explained by missed appointment or loss of pills. The second reason was being away from home, mainly due to the economic activities in the area. In contrary to previous studies, feeling better or drugs side effects were not frequently reported as reasons for non adherence [35]–[37].

This cross-sectional study has several limitations: i) This design allows a rapid assessment easily replicated in other programmes or other time periods but can only measure adherence at one point of time and does not allow assessing the variation of adherence over the full length of treatment compared to the use of a prospective cohort study [38]. ii) Twenty four percent of the eligible survey population were not assessed for adherence. The exclusion of patients who defaulted before the survey was related to the use of a cross-sectional design and resulted in an overestimation of the adherence. Nevertheless, even when adherence to treatment was estimated including the defaulters and considering them as non-adherent, the adherence level remained fairly good, close to 80%. Similarly, some patients were not interviewed because either they died before the survey; were hospitalised at the time of the survey; could not be located or refused to consent for an home visit. iii) Adherence was assessed among patients who had come recently to collect their pills at the TB clinic. This may have overestimated the adherence because treatment adherence is likely to be better in the days surrounding clinical appointment [14]. iv) Another limitation was the high number of missing values for the pill count. This is mostly explained by the fact that, for several patients, it was difficult to know exactly how many pills they have received at the last appointment to the TB clinic, and that some surveyors, at the beginning of the survey, included erroneously the pyridoxine in the TB pill count. v) Finally, self-reported adherence measures are prone to social desirability bias as patients might tend to provide answers that would fit the surveyors expectations (that is, good adherence to treatment). We tried to limit this bias by working with surveyors who were not part of the staff providing care to the patient.

Since there is no gold standard to assess adherence to TB treatment, it is suggested to use a combination of adherence monitoring tools [15]. The Medication Event Monitoring System (MEMS®) is an objective tool often used in studies on treatment adherence [14]. It is an electronic device included in a drug container, which records the date and hour of each opening. However, this device is very technical, it is expensive and does not ensure that the patient actually ingested the pill. For these reasons as well as for operational reasons, we decided not to use it in this survey. We used a combination of objective (INH urine test, pill count) and subjective tools (VAS and questionnaire). Studies using pill count, VAS and questionnaires in monitoring adherence to HIV treatment have reported good agreement between questionnaires and VAS [17], [19], pill count and VAS [16], [19] or questionnaire [18], [19], and between different measures of self-reported adherence [20], [21]. We expected similar results but the observed agreement between the adherence measurement tools used in this survey was low (kappa≤0.43) as well as most of the estimated maximum attainable kappa. The kappa coefficients should be interpreted with caution as confidence intervals were wide, the sample of patients selected for adherence assessment was likely to be more homogeneously adherent, and the low prevalence of non-adherence [30] might have influenced the magnitude of the coefficients. Nevertheless, this low agreement could also be explained by the fact that each adherence measurement tool was measuring different components of adherence, over different time periods, which gives another reason to combine different tools to monitor adherence.

In the absence of gold standard to measure adherence to TB treatment, we decided to use a LCA model. In this model, the combination of two out of three adherence measurement tools (INH test, questionnaire, VAS) predicted very well the adherence to TB treatment. Due to the number of missing values, it was not possible to include the pill count in the model.

INH urine test is the most objective tool for monitoring of adherence to TB treatment. However, it only reflects recent dose intake and, therefore, if performed in the health facility, INH urine test might overestimate the adherence level of patients as they may tend to ingest pills just before their visit. Also, the relatively high price, the supply constraints and the storage condition (cold chain) of this manufactured test make it difficult to use in routine conditions. Although these limitations may be partly overcome by the possibility of making local non commercial tests for a much cheaper cost [39], [40], a urine test may appear intrusive for the patient and might not be suitable to monitor adherence routinely under program conditions. In addition, pill count has been shown to be a reliable tool for monitoring of adherence to HIV treatment [18]. However, the accuracy of a clinic-based planned pill count may be easily distorted by the patient and is limited by patients failing to bring all their pills to the clinic. Also, as the INH test, pill count appears contradictory to patient's empowerment. On the contrary, questionnaires and VAS have been described to be easy to use, non intrusive and cheap tools to measure both recent and last month adherence [19]. Pictographic and color VAS have been shown to be valid and useful tools in assessing medication adherence in lower-literacy populations [41]. Thus, these tools seem to be well adapted to programmatic conditions after training on their use.

In conclusion, this survey conducted in routine program conditions has shown that self-administered therapy together with the FDC and patient centred adherence strategies allows achieving appropriate adherence to antituberculosis treatment in a high TB and HIV burden area. This strategy is well adapted to limited resources settings. However, these results can not be directly extrapolated in settings where single antituberculosis drugs are administered separately. Although the use of a combination of two simple tools, such as the VAS and a questionnaire, might be an adequate approach to monitor adherence to TB treatment in routine program conditions, further validation is required. Also, in the future, other tools might play a role in the support and monitoring of adherence to TB treatment, in particular communication devices such as mobile phones, which are more and more available in high burden and limited resource countries [42], [43].
